# A Review of 3-Nitrooxypropanol for Enteric Methane Mitigation from Ruminant Livestock

**DOI:** 10.3390/ani11123540

**Published:** 2021-12-13

**Authors:** Guanghui Yu, Karen A. Beauchemin, Ruilan Dong

**Affiliations:** 1 College of Animal Science and Technology, Qingdao Agricultural University, No. 700 Changcheng Road, Chengyang District, Qingdao 266109, China; guanghuiyu@qau.edu.cn; 2 Lethbridge Research and Development Centre, Agriculture and Agri-Food Canada, 5403 1st Avenue South, Lethbridge, AB T1J 4B1, Canada; karen.beauchemin@agr.gc.ca

**Keywords:** enteric methane, hydrogen production, methanogens, mitigation, ruminant livestock, 3-nitrooxypropanol

## Abstract

**Simple Summary:**

Enteric methane (CH_4_) from the anaerobic fermentation of feed carbohydrates in ruminant livestock accounts for 3 to 5% of global greenhouse gas emissions. Among the different CH_4_ mitigating approaches evaluated to decrease enteric CH_4_ emissions from ruminants, the feed additive 3-nitrooxypropanol is effective with a mean reduction in CH_4_ of 30%, depending on animal type, diet and dose. 3-nitrooxypropanol is chemically synthesized and studies show low safety risk with no detrimental effects to animals and humans. 3-nitrooxypropanol was recently approved by regulatory authorities for use in Brazil and Chile and has received a favorable opinion from the scientific panel of the European Food Safety Authority, with approvals in various jurisdictions expected in the near future. As a substantial body of research on 3-nitrooxypropanol is now available, this review offers a timely analysis of the opportunities and challenges of using 3-nitrooxypropanol to mitigate enteric CH_4_ emissions in ruminant livestock.

**Abstract:**

Methane (CH_4_) from enteric fermentation accounts for 3 to 5% of global anthropogenic greenhouse gas emissions, which contribute to climate change. Cost-effective strategies are needed to reduce feed energy losses as enteric CH_4_ while improving ruminant production efficiency. Mitigation strategies need to be environmentally friendly, easily adopted by producers and accepted by consumers. However, few sustainable CH_4_ mitigation approaches are available. Recent studies show that the chemically synthesized CH_4_ inhibitor 3-nitrooxypropanol is one of the most effective approaches for enteric CH_4_ abatement. 3-nitrooxypropanol specifically targets the methyl-coenzyme M reductase and inhibits the final catalytic step in methanogenesis in rumen archaea. Providing 3-nitrooxypropanol to dairy and beef cattle in research studies has consistently decreased enteric CH_4_ production by 30% on average, with reductions as high as 82% in some cases. Efficacy is positively related to 3-NOP dose and negatively affected by neutral detergent fiber concentration of the diet, with greater responses in dairy compared with beef cattle when compared at the same dose. This review collates the current literature on 3-nitrooxypropanol and examines the overall findings of meta-analyses and individual studies to provide a synthesis of science-based information on the use of 3-nitrooxypropanol for CH_4_ abatement. The intent is to help guide commercial adoption at the farm level in the future. There is a significant body of peer-reviewed scientific literature to indicate that 3-nitrooxypropanol is effective and safe when incorporated into total mixed rations, but further research is required to fully understand the long-term effects and the interactions with other CH_4_ mitigating compounds.

## 1. Introduction

Methane (CH_4_), a flow gas, is a potent greenhouse gas with a global warming potential 82 times stronger per unit mass than carbon dioxide (CO_2_) on a 20-year timescale and 28 times more powerful on a 100-year time scale [1]. CH_4_ emissions from enteric fermentation of plant biomass in the ruminant digestive system generated by methanogenic archaea not only contribute to climate change, but also represent a loss of 2 to 12% of gross energy intake and a potential reduction in feed efficiency [2]. Enteric CH_4_ from ruminant livestock escapes into the atmosphere mainly through eructation, and contributes 3 to 5% of the global greenhouse gas emissions [3]. The world’s increasing demand for animal-sourced protein products will undoubtedly cause enteric CH_4_ emissions to increase [4] unless mitigation is adopted. According to Rogelj et al. (2018) [5], CH_4_ emissions from agricultural production need to be reduced by 24 to 47% by 2050 relative to 2010 to meet the 1.5 °C target of the Paris Agreement [6]. Over 100 countries (including 9 of the world’s top 20 CH_4_-emitting countries) recently signed a pledge to reduce global CH_4_ emissions by at least 30% relative to 2020 levels by 2030 [7]. CH_4_ has an estimated lifetime of 12 yr in the atmosphere [8], hence decreasing global CH_4_ emissions can limit global climate warming in a short timeframe.

Given the global emphasis on CH_4_ reduction, numerous mitigation strategies have been studied. These include dietary formulation [9], animal breeding [10], vaccines [11], bromoform-containing seaweeds [12], chemical inhibitors [13], and others. Despite research efforts, few technologies are commercially available that can safely, consistently, and substantially reduce enteric CH_4_ from ruminant livestock_._ Diet formulation typically results in only moderate reductions in CH_4_ (<20%), breeding for low-CH_4_ emitting animals may bring moderate reductions but requires a long term approach, vaccines against methanogens are at a developmental stage, and given that bromoform is a potential carcinogen the safety risks associated with *Asparagopsis* sp. seaweeds [12] may limit their extensive use in animal diets. Numerous chemical CH_4_ inhibitors have been evaluated over the years, and while some have been shown to be highly effective, achieving large reductions in CH_4_ emissions (>30%), their commercial use has been limited mainly due to safety concerns. One notable exception is 3-nitrooxypropanol (3-NOP), which has been shown in the past decade to be highly effective in decreasing CH_4_ production while posing minimal safety risk. 3-NOP binds to the CH_4_-producing enzyme methyl-coenzyme M reductase (MCR), thereby inhibiting the formation of CH_4_ without negative influence on non-methanogenic bacteria or the animal itself [14,15].

Feed additives that persistently lower CH_4_ emissions must not have toxic effects for animals, humans and the environment. To be adopted by producers, they need to be easy to use and preferably low cost. An increase in animal productivity would help offset the additional cost of the feed additive and improve profitability [9]. 3-Nitrooxypropanol has been evaluated in approximately 28 in vivo and 7 in vitro ruminant studies and several recent meta-analyses have examined this substantial body of information to examine overall efficacy when 3-NOP is used for enteric CH_4_ mitigation [16,17,18,19,20,21]. 3-NOP could provide a feasible strategy for CH_4_ mitigation if it is accepted by consumers and approved by regulatory authorities. 3-NOP recently received a favorable opinion from the scientific panel of the European Food Safety Authority for safety and efficacy in dairy cows. It was recently approved in Brazil and Chile, and regulatory approvals in other jurisdictions are expected in the future. Thus, with impending on-farm use of 3-NOP, there is a need to critically examine the body of information available to enable farmers and technical advisors to make informed decisions. This review provides a comprehensive analysis of the published results and discusses the challenges and opportunities for using 3-NOP to reduce enteric CH_4_ emissions from ruminant livestock.

## 2. 3-Nitrooxypropanol, Mode of Action and Safety

**Figure 1:**
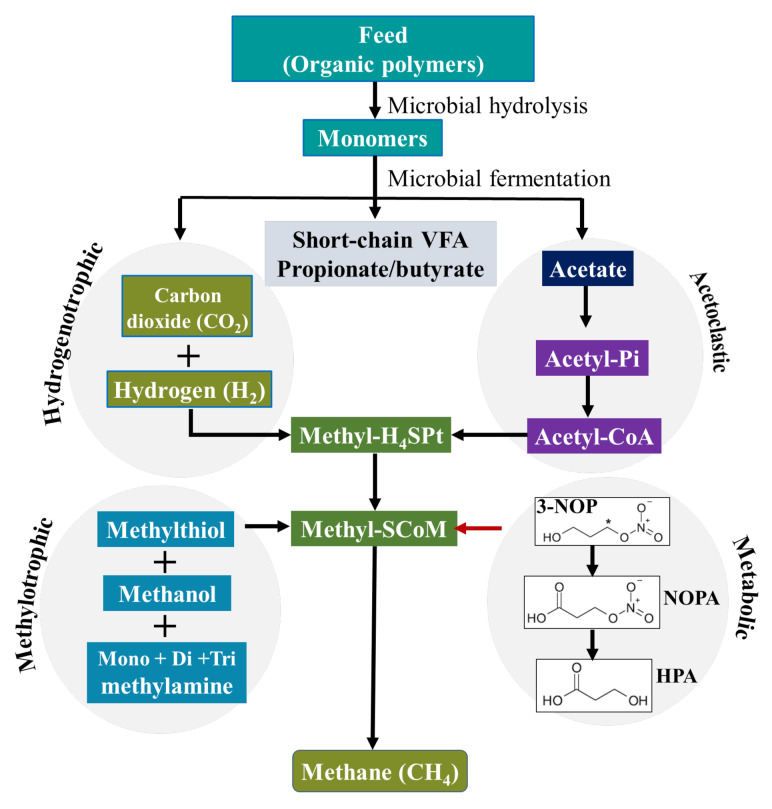
The main CH_4_ formation pathway in the rumen of ruminants and its inhibition by 3-NOP [28,29]. (3-NOP = 3-nitrooxypropanol; NOPA = 3-nitrooxypropionic acid; HPA = 3-hydroxypropionic acid).

The compound 3-NOP was first chemically synthesised by Ogawa et al. (1990) [22], and a patent was granted for the use of 3-NOP as a CH_4_ mitigant [23]. It has low molecular weight (121.09 g/mol) and is a small molecule with dual chemical functional groups: a primary alcohol and an organic nitrate ester [24]. The nitrogen (N) atom is indirectly attached to the carbon (C) backbone via a C–O–N bond (chemical structure shown in Figure 1). As a structural analogue of methyl-coenzyme M, 3-NOP specifically targets the nickel enzyme MCR [15].

Due to its molecular structure, 3-NOP is highly soluble and rapidly metabolized in the rumen to very low concentrations of nitrate, nitrite and 1,3-propanediol. Duin et al. (2016) [15] reported that 3-NOP is hydrolyzed in rumen fluid to 1,3-propandiol, a compound of low toxicity, which is further transformed into 3-hydroxypropionic acid (HPA) [25]. Thiel et al. (2019) [24] demonstrated that 3-NOP is first oxidized to 3-nitrooxypropionic acid (NOPA), which is then hydrolyzed to HPA and inorganic nitrate. In ruminants, NOPA is a plasma metabolite and HPA is a compound of naturally occurring intermediary metabolism. HPA is further used by mammalian cells as substrate for synthesis of acetyl-CoA and propanoyl-CoA. The latter serves as substrate for gluconeogenesis and is beneficial for lactating ruminants because propanoyl-CoA is a prominent carbon source [24].

The molecular shape of 3-NOP is similar to that of methyl-coenzyme M, a co-factor involved in methyl transfer during methanogenesis. Duin et al. (2016) [15] showed that 3-NOP specifically binds into MCR and inactivates the enzyme by temporarily oxidizing the nickel ion from oxidation state (+1) to (+2) in the active site, leading to an inhibition of methanogenesis. MCR is a nickel enzyme in which the nickel is bound in a tetrapyrrole derivative named cofactor F430 [26]. This nickel-containing cofactor has to be in the Ni(I) oxidation state for the enzyme to be active to catalyze the CH_4_-forming step in rumen fermentation. The moderate oxidation potential of 3-NOP makes it inactivate MCR at micromolar concentrations. Duin et al. (2016) [15] showed that 3-NOP preferably targets the active site of MCR in a pose that places its reducible nitrate group in electron transfer distance to Ni(I). Thus, the inhibition of CH_4_ formation during the last step of the methanogenesis pathway in rumen methanogenic archaea is achieved (Figure 1).

Residues in milk and meat are minute or non-existent and the safety risks of 3-NOP are seemingly low [24,27]. It is reported that 3-NOP and its metabolites pose no mutagenic and genotoxic potential [27]. Although neither ^14^C-3-NOP nor ^14^C-NOPA were found in milk [24], further studies over a range of animals and diets are required to confirm the absence of 3-NOP residues in manure, meat or milk to address food safety concerns.

## 3. Effects on Rumen Fermentation and Methanogenesis

Plant material consumed by ruminants is degraded in the anaerobic environment of the rumen by bacteria, protozoa, and fungi predominantly yielding volatile fatty acids (VFA), CO_2_, NH_3_, and CH_4_ with hydrogen (H_2_) as intermediate [30]. The VFA (mainly acetate, propionate, butyrate) are metabolized and absorbed as the primary source of energy for ruminant animals, whereas CH_4_ is formed by methanogenic archaea from CO_2_ and H_2_. Hence, enteric CH_4_ is a by-product of the normal fermentation process of feed in the rumen and hindgut of ruminant livestock and it is the main H_2_ sink in the rumen. Methanogenesis is a pathway to generate energy for methanogenic archaea [16], whereby MCR, a unique enzyme found in archaea, catalyzes methyl-coenzyme M and coenzyme B to CH_4_ during the last step of methanogenesis [15].

There are various ways in which 3-NOP affects fermentation and methanogenesis. As a structural analogue of methyl coenzyme M, 3-NOP acts as a competitive inhibitor that selectively binds to and targets the active site of MCR [15], as discussed previously. As a result of inhibiting CH_4_ formation using 3-NOP, the fermentation pathways are shifted towards alternative H_2_ sinks such as propionic acid production [16,31]. Most studies consistently report increased propionate proportions at the expense of acetate proportions in rumen fluid with feeding of 3-NOP [14,32,33]. A recent meta-analysis [19] showed that increasing levels of 3-NOP supplementation in dairy diets linearly decreased proportion of acetate and increased that of valerate. In the same meta-analysis but using a beef cattle database, the total VFA concentration and the proportion of acetate were significantly decreased with increasing 3-NOP supplementation, whereas other individual VFA increased [19]. A change in the end-products of rumen fermentation when feeding 3-NOP can have important consequences for animal metabolism. Acetate is metabolized by peripheral tissues and other organs of the portal-drained viscera and completely oxidized to CO_2_ entering the Krebs cycle to supply energy or used for milk fatty acid synthesis in ruminants, with low proportion absorbed in the rumen epithelium for the formation of ketone bodies [34]. Propionate is metabolized by the liver, which may enter the Krebs cycle to be totally oxidized to CO_2_ or to produce lactate, pyruvate, and alanine and then entering the gluconeogenesis pathway to synthesize glucose or glycogen, and may also be used as a source of carbon skeleton for new cell synthesis [35]. Ruminal butyrate proportion also tends to increase with 3-NOP supplementation of diets, with butyrate absorbed through the rumen wall and mostly metabolized by rumen epithelial cells as an energy source or converted into β-hydroxybutyrate [36,37].

H_2_ is used as a substrate by methanogenic archaea to generate energy and this process is decreased in the presence of 3-NOP. Inhibiting methanogenesis can cause dissolved H_2_ to accumulate in the rumen, and if not totally incorporated into other H_2_ sinks (e.g., formate, propionate, valerate, caproate, ethanol, lactate, microbial protein and fatty acid synthesis), the H_2_ gas is expelled from the rumen [38,39] representing a loss of energy. Thus, gaseous H_2_ emissions can increase in animals receiving 3-NOP [14,36,38].

3-NOP has been shown to have limited effects on the growth characteristics of rumen protozoa and bacteria when tested in vivo and in vitro [37,40,41], but populations of methanogenic archaea were decreased [16]. 3-NOP has also been shown to inhibit abundance of hydrogenotrophic methanogens in some studies [42]. Abundances of methanogens (5.6-fold), *Methanomassiliicoccaceae* family (4-fold), and *Methanobrevibacter* (5.6-fold) in rumen pellet samples were decreased with 3-NOP addition compared with the control [43]. Pitta et al. (2021) [42] reported differential responses among methanogens in dairy cows receiving 60 mg 3-NOP/kg DM; *Methanobrevibacter* was reduced at week 4, *Methanobrevibacter ruminantium was* reduced from week 8, and *Methanosphaera was* reduced at weeks 8 and 12. Dosing 200 mg 3-NOP/DM to beef cattle significantly decreased abundances of *Methanobrevibacter*, *Methanomicrobium*, and *Methanomethylophilus* in both rumen fluid and digesta [44]. In addition, the effect of 3-NOP on methanogens depends upon the diet, as Zhang et al. (2020) reported 3-NOP decreased the abundance of *Methanobrevibacter* in cattle fed barley silage, but not when fed grass hay [45].

Most studies showed no effect of 3-NOP on ammonia N concentration, except when a high level of 3-NOP was used [16,37,46]. In the meta-analysis of Jayanegara et al. (2018) [16], addition of 3-NOP increased rumen pH (pH = 0.56 (±0.13) × 3-NOP (g/kg DMI) + 6.40 (±0.05) (R^2^ = 0.69, n = 14, *p* < 0.01)), although Haisan et al. (2017) [41] and Lopes et al. (2016) [46] reported no effects of 3-NOP on ruminal pH. An increase in rumen pH may be related to the observed increase in feeding frequency of animals consuming 3-NOP compared to control [47]. It may also be related to decreased DMI, decreased total VFA concentration and increased butyrate molar percentage and uptake from the rumen [48].

## 4. Mitigation of Enteric CH_4_ Using 3-Nitrooxypropanol

### 4.1. Method of Providing 3-Nitrooxypropanol to Animals

Use of 3-NOP for CH_4_ mitigation has been evaluated in animals in confinement, with no published research with grazing animals. Various methods of providing 3-NOP to ruminant livestock have been used: 3-NOP delivered directly into the rumen at feeding time [33], top dressed onto feed in a manger [40], mixed into a total mixed ration (TMR) [49], incorporated into a concentrate pellet [50], and added to the roughage component [50]. 3-NOP was shown to be effective when added to the TMR or a component of the ration, but the mitigation effect when dosing it into the rumen was transitory indicating the product may rapidly leave the rumen in the liquid outflow. Incorporating 3-NOP into a ration or a component of the ration (concentrate, forage), appears to lead to a more continuous presence in the rumen as animals consume their feed throughout the day [14]. Several studies have also shown that once 3-NOP is removed from the diet, its effect on CH_4_ is negated within several days [49,51].

### 4.2. Efficacy and Uncertanty

Inclusion of 3-NOP in ruminant diets decreases enteric CH_4_ emissions in a dose–response manner [16,17,19]. In the meta-analysis of Dijkstra et al. (2018) [17] from 11 studies, the average 3-NOP dose used in beef cattle was 144 mg/kg of DM, ranging from 50 to 345 mg/kg of DM; in dairy cattle, the average dose was 81 mg/kg of DM, ranging from 27 to 135 mg/kg of DM. An intermediate 3-NOP dose (111.2 mg/kg DM) was evaluated in a sheep study [52].

Several meta-analyses report that increasing dosage level of 3-NOP linearly decreased enteric CH_4_ emissions (Table 1). When enteric CH_4_, expressed relative to digested organic matter (DOM) or DMI, was regressed against dietary 3-NOP dose (mg/kg of DM), the R^2^ was relatively high [16]. In addition, Romero-Perez et al. (2014) [40] reported a linear effect of 3-NOP dose (47, 144 and 305 mg/kg DM) on total CH_4_ emissions (g/d) per animal. Vyas et al. (2016) [53] also reported a linear effect of 3-NOP dose between 100 and 200 mg/kg DM on CH_4_ yield (g/kg DMI, maximum decrease of 45%) in feedlot cattle. In mid- to late- lactation dairy cows, Hristov et al. (2015) [14] observed a linear effect of 3-NOP dose from 40 to 80 mg/kg DM on enteric CH_4_ emission (g/d). Melgar et al. (2020) evaluated 6 levels of inclusion of 3-NOP (40, 60, 80, 100, 150, and 200 mg/kg of feed DM) in dairy cows and observed a linear effect of 3-NOP dose (with maximum mitigation effect at 150 mg/kg but with no statistical difference among 100, 150, and 200 mg/kg). In contrast, no linear response to 3-NOP concentration was observed in beef cattle by Alemu et al. (2021) [38,54], for reasons that are not clear.

When examined across published studies, the efficacy of 3-NOP in decreasing CH_4_ emissions was greater in dairy cattle (R^2^ = 0.92) compared with beef cattle (R^2^ = 0.80) [17,19], when compared at the same dose. Based on the meta-analysis by Kim et al. (2020) [19], dosing 100 mg 3-NOP/kg DMI would be predicted to decrease enteric CH_4_ emissions in dairy cattle by 36.4% compared with 17.3% in beef cattle. This difference between cattle type is confounded by the types of diets and level of DMI in these studies. According to equations in the meta-analysis by Kim et al. (2020) [19], a dose of 60 to 80 mg 3-NOP/kg DMI for dairy cows and 150 to 200 mg 3-NOP/kg DMI for beef cattle would be expected to decrease enteric CH_4_ production by 30%. In a meta-analysis, Dijkstra et al. (2018) [17] showed that in addition to 3-NOP dose, type of animal and nutrient composition of the diet explained most of the variability in 3-NOP response. An increased neutral detergent fiber concentration of the diet was shown to negatively affect the anti-methanogenic effect of 3-NOP (10 g/kg DM increase in dietary neutral detergent fiber lowers the efficacy of 3-NOP to decrease CH_4_ production by 1.64 ± 0.33%) [17]. Therefore, in the same cattle type, the mitigation effect of 3-NOP has been greater in high concentrate diets [40,51,55] and less in high fiber diets [17,38]. For example, several studies using 3-NOP as a feed additive have reported very high reductions in CH_4_ emissions from feedlot cattle fed grain-based diets (82% in Vyas et al. (2016) [51] and 80% in McGinn et al. (2019) [56]). Other factors causing variability in response to 3-NOP may be related to method used to measure CH_4_ emissions (chambers, sulfur hexafluoride tracer technique, and Greenfeed system), duration that cattle were fed 3-NOP (short- vs. long-term), and interaction effects when 3-NOP was combined with other mitigation strategies (e.g., monensin [55], unsaturated fatty acids [57], higher concentrate proportion [37], and others; Table 2 and Table 3).

### 4.3. Effectiveness of 3-Nitrooxypropanol in Long-Term Studies

In 5 long-term experiments (defined as 10-week [50], 12-week [14], 15-week [55], 16-week [49], and 34-week [51] feeding periods), CH_4_ yield (g/kg DM) was significantly linearly (R^2^ = 0.91, n = 19, *p* < 0.01) decreased with increasing level of 3-NOP addition [19]. Thus, it appears that overall, the responses in long-term studies have been generally similar to those observed in short-term studies. Hristov et al. (2015) [14] reported 30% less CH_4_ (g/d) on average for lactating dairy cows fed 40 to 80 mg 3-NOP/kg DMI over 12 weeks. In 10- [50] and 15-week [60] experiments with dairy cattle, CH_4_ (g/d) decreased on average over the study by 26 to 28% with 3-NOP (40 to 80 mg/kg feed DMI), and this effect did not diminish over time. Romero-Perez et al. (2015) [49] reported adding 3-NOP to a beef cattle diet for 16 weeks resulted in a sustained reduction in enteric CH_4_ emissions (59%; 9.16 vs. 22.46 g/kg DMI), with no decline in response when measurements were repeated over time. In a 34-week feeding study by Vyas et al. (2016) [51], supplementation of 3-NOP at 200 mg/kg DM decreased, on average, emission of enteric CH_4_ (g/d) by 82% in feedlot finishing beef cattle with the effect negated within days once 3-NOP supplementation was discontinued. However, in a beef cattle feedlot study by McGinn et al. (2019) [56], there was a small, constant decline in CH_4_ emission reduction (from 80% to 60% reduction over 90 d using micrometeorological methods), indicating a possible adaptation of the rumen microbiome. Similarly, in a dairy cattle study by Melgar et al. (2020) [36], the CH_4_ mitigation effect of 3-NOP decreased over 15 weeks. Alemu et al. (2021) [54] reported a 22% reduction in efficacy of 3-NOP to decrease CH_4_ yield (g/kg DM) in beef cattle when a low dose (100 mg/kg DMI) was fed for 16 weeks, but no reduction in efficacy occurred over time when higher doses were used (125 to 150 mg /kg DMI). Nonetheless, other studies have shown no decline in the effectiveness of 3-NOP over time [14,38,49]. It is evident that further research is needed to determine whether the response to 3-NOP is maintained over the long-term. Studies with repeated measurements over the feeding period and over multiple years for mature beef cows and over multiple lactations for dairy cows are needed to ensure the mitigation effect of 3-NOP is persistant. This is an important aspect given that the potential for adaptation of the rumen microbiome such that compounds diminish in effectiveness has been shown with other rumen modifying compounds (e.g., ionophores [69], essential oils [70], etc.).

## 5. Effects of 3-Nitrooxypropanol on Dry Matter Intake, Digestibility and Animal Productivity

### 5.1. Effects of 3-Nitrooxypropanol on Dry Matter Intake and Digestibility

The effects of 3-NOP on DMI appear to be different among studies and may depend on dose, animal type, diet and the duration of feeding [54]. In the meta-analysis of Kim et al. (2020) [19] from 14 beef cattle studies, DMI tended to decrease (slope = −0.0016, *p* = 0.06, and *R*^2^ = 0.17) as the dose of 3-NOP supplemented increased. However, using a dairy cattle database, Kim et al. (2020) [19] reported that 3-NOP supplementation had no significant linear relationship with DMI. Using a combined beef and dairy cattle database, Kim et al. (2020) [19] reported that increasing the dose of 3-NOP supplementation linearly decreased DMI (slope = −0.0017, R^2^ = 0.17, *p* < 0.05). However, in the meta-analysis of Jayanegara et al. (2018) [16] from 12 studies, DMI from ruminants (dairy cows, beef cattle, and sheep) was not linearly decreased with increasing level of 3-NOP addition.

Inconsistent effects of 3-NOP on DMI between beef and dairy cattle studies may be due to the higher doses of 3-NOP used in most beef studies. For example, in dairy cattle studies that used a dose of 40 to 80 mg 3-NOP/kg DM, DMI was not affected [14,31,46]. However, in a beef cattle study with doses of 47, 144 and 305 mg/kg DM, a linear decrease in DMI was reported [40]. Additionally using relatively high doses of 3-NOP (200 mg/kg DM) in beef cattle fed backgrounding diets, Vyas et al. (2016) [51] and Vyas et al. (2018) [55] reported 8% and 7% reductions in DMI, respectively, compared with control animals. Alemu et al. (2021) [54] reported an initial reduction in DMI (kg/d) of 6.1% and 6.4% for feedlot finishing diets fed 100 and 150 mg 3-NOP/kg DM, respectively, but after 56 days of consuming 3-NOP, there was no difference in DMI between treatment and control cattle. This trend may indicate an adaptive response of the cattle over time. The decrease in DMI with the higher doses of 3-NOP typically fed in beef studies might be due in part to palatability effects [54]. In addition, the high starch concentration of beef cattle finishing diets results in a rumen fermentation with greater molar proportion of propionate, compared with dairy cattle. A further increase in molar proportion of propionate with feeding of 3-NOP may augment the hyperphagic effect of absorbed propionate causing DMI to decline [71]. Other factors may be related to chemical composition and particle size of the diet, and silage fermentation products [71].

In earlier work, it was assumed that inhibiting methanogenesis would decrease diet digestibility. Methanogenesis is the main route of cofactor re-oxidation in the rumen and when inhibiting methanogenesis, elevated H_2_ concentration can hinder cofactor re-oxidation and thus inhibit fermentation [72]. Reduced co-factors need to be re-oxidized in the rumen for fermentation to continue. However, studies have shown no adverse effects of 3-NOP on diet digestibility in beef cattle [40], early-lactation dairy cows [31], or in specific breeds of cattle (Friesian Holstein, Angus and Segurena breeds) [16]. Additionally, a relatively small increase in apparent total-tract digestibility of several nutrients upon feeding 3-NOP was reported in some studies. These include DM [14,31,41], organic matter [31], crude protein [14,36,57], neutral detergent fiber [31,41], acid detergent fiber [14], gross energy [31] and starch [57]; however, these small improvements in digestibility are not likely to affect animal performance.

### 5.2. Effects of 3-Nitrooxypropanol on Animal Productivity

Improvements in animal performance when supplementing diets with CH_4_ would help incentivize producers to adopt such a technology [14,19]. Theoretically, a decrease in CH_4_ production could provide more metabolizable energy intake for productive purposes, such as milk production or growth if DMI is not proportionally decreased, and the shift in ruminal fermentation end-products are in a form that could be used as energy substrates [73]. In the meta-analysis of Jayanegara et al. (2018) [16], increasing the level of 3-NOP in beef cattle diets significantly improved gain to feed ratio (slope = 0.05, *p* < 0.01, and *R*^2^ = 0.94) and did not show any adverse effects on average daily gain. Using a dairy cattle database, addition of 3-NOP increased milk fat concentration (slope = 1.5, *p* < 0.05, and *R*^2^ = 0.47) and tended to increase milk protein concentration, whereas other lactation performance characteristics were not affected by addition of 3-NOP [16]. In the meta-analysis of Kim et al. (2020) [19], 3-NOP supplementation of dairy diets tended to increase milk fat and milk protein and decrease milk yield, but 3-NOP had no effect on fat corrected milk or milk lactose percentage. Ungerfeld (2018) [18] also reported no relationship between inhibiting methanogenesis and DMI-adjusted energy corrected milk production.

When examining individual beef cattle studies, supplementing 3-NOP to finishing cattle improved gain-to-feed ratio by 3% [55], with no adverse effects on weight gain [61]. In dairy cattle studies, feeding 3-NOP increased milk protein (g/100 g of milk) [31,33] and milk fat content (g/100 g of milk) by up to 8% [31,46]. Improvements is milk quality (milk fat and milk protein) may have resulted from a slight increase in net energy intake for lactation due to the decrease in feed energy lost as CH_4_ or the shift in fermentation end-products towards increased propionate synthesis. Schilde et al. (2021) [37] reported an energy corrected milk yield (kg/d) reduction of 8.8% with inclusion of 3-NOP (60 mg 3-NOP/kg DM) in high concentrate feed as compared with the control without 3-NOP in cows from parturition until d 120 postpartum. Many studies indicate that feeding 3-NOP to dairy cows did not affect milk yield [14,31,32,33,41,50,60], although there is a lack of large-scale long-term studies.

## 6. Practical Considerations for Use on Farm

While the extensive body of published literature under controlled research conditions indicates that 3-NOP consistently decreases CH_4_ production from ruminant livestock by on average 30%, it is important to state that many of these studies are short-term and even the long-term studies have been limited to several months in duration. No published study has examined the effects of feeding 3-NOP over multiple lactations or seasons, which is important information for farmers. 3-NOP has been used in commercial conditions [38,54], but further information on using 3-NOP under a broad range of feeding systems is still needed. Furthermore, the use of 3-NOP on-farm as a feed additive requires regulatory approval, which has been granted thus far in Brazil and Chile.

When using a CH_4_ mitigation strategy it is important to ensure that emissions elsewhere in the supply chain are not inadvertently increased. Thus, the impact of using 3-NOP for enteric CH_4_ mitigation on other emissions, such as manure CH_4_ emissions also need to be considered. Nkemka et al. (2019) [74] showed no residual effects of feeding 3-NOP to beef cattle on manure CH_4_ emissions when the manure was used in an anaerobic digester. Owens et al. (2020) [75] showed no residual effects of feeding 3-NOP to beef cattle on greenhouse gas (CH_4_, CO_2_ and nitrous oxide) emissions from manure decomposition during storage. However, in a laboratory scale study using soils amended with manure from cattle fed 3-NOP, Weber et al. (2021) [76] showed that GHG emissions were dependent on soil texture. For coarse-textured soil (Black Chernozemic), GHG emissions were greater when amended with manure from cattle fed 3-NOP compared with control manure (mainly due to increased nitrous oxide emissions), but this effect was not observed for other soil types or when the manure was first composted. The possible carryover effects of feeding 3-NOP on manure CH_4_ emissions needs further study.

In addition, the emissions from producing 3-NOP need to be included when evaluating the net impact on total greenhouse gases, even though CO_2_ emissions from manufacturing 3-NOP are very small in comparison to the decrease in CH_4_ production. The emission factor for 3-NOP was reported as 47.9 kg CO_2_e/kg 3-NOP in the study by Alvarez-Hess et al. (2019) [77] and 52 kg CO_2_e/kg 3-NOP in the study by Kebreab and Feng (2021) [78]. Thus, dosing 60 mg 3-NOP/kg DMI to dairy cattle and 150 mg 3-NOP/kg feed DMI to beef cattle, respectively would represent approximately 3 g CO_2_e/kg feed DM (equivalent to 0.1 g CH_4_) in dairy cattle compared with 8 g CO_2_e/kg feed DM (equivalent to 0.3 g CH_4_) in beef cattle [78].

At present, use of 3-NOP is limited to confinement non-organic systems using formulated diets, as it needs to be fed as part of the ration. Globally, it is estimated that 37% of enteric CH_4_ emissions from ruminant livestock production is pasture-based [79], and thus a significant proportion of ruminant farming is currently excluded from the potential for mitigation using 3-NOP in its present form. However, research is ongoing to extend its application under grazing conditions [20]. This may include adding 3-NOP to pasture supplements, use of lick blocks, encapsulation, slow-release ruminal devices, and so forth. At present, little is known of the effectiveness of 3-NOP for grazing ruminants. Another method of using 3-NOP has been to administer it to neonatal animals, a concept referred to as early life programming. The central idea is that the developing microbial community of the newborn ruminant is more malleable than that of the adult ruminant and that its manipulation could have long-lasting effects. In a study by Meale et al. [59], 3-NOP was dosed daily (3 mg 3-NOP/kg BW) to calves from birth until 14 weeks (3 weeks after weaning), and a 12% reduction in CH_4_ emissions (g/d) was observed for 9 weeks after 3-NOP dosing was discontinued. Furthermore, a reduction in CH_4_ was noted when measured almost a year later. While early life intervention to decrease CH_4_ emissions is still at an early stage, it remains a possibility for future application and may have potential especially for grazing ruminants where delivery mechanisms for 3-NOP are limited.

## 7. Conclusions

In conclusion, there is overwhelming scientific evidence that incorporation of 3-NOP in the diets of ruminant livestock inhibits enteric CH_4_ emissions in a dose-dependent manner without negative effects on animal production. Safety risks for animals and humans appear to be minimal. Overall effects on animal productivity are small, albeit positive, with improvements in milk quality (milk fat and milk protein) in some dairy cattle studies and feed conversion efficiency in some beef cattle studies. Multi-year published studies are needed to determine the long-term impacts of using 3-NOP for CH_4_ mitigation and further research is required to explore practical use of 3-NOP for grazing animals. If approved by regulatory authorities, use of 3-NOP in ruminant diets represents a significant advance in terms of offering livestock producers a practical means of lowering CH_4_ emissions.

## Figures and Tables

**Table 1:** Linear relationships between enteric CH_4_ and dose of 3-NOP (g/kg DM [16], mg/kg DM [17,19]) in ruminant diets.

Type ^1^	Equation ^2^	Source
all	CH_4_/DMI (g/kg DMI) = −38.7 (±6.3) × 3-NOP + 20.2 (±1.25) (R^2^ = 0.59, n = 39, *p* < 0.01)	[16]
all	CH_4_/DMI (g/kg DMI) = −0.00158 (±0.000544) × 3-NOP + 12.3 (*p* < 0.05)	[17]
all	CH_4_/DMI (g/kg DMI) = −0.041 (±0.0047) × 3-NOP + 20.636 (±1.02) (R^2^ = 0.74, n = 54, *p* < 0.01)	[19]
beef	CH_4_/DMI (g/kg DMI) = −0.037 (±0.0043) × 3-NOP + 21.365 (±1.48) (R^2^ = 0.80, n = 35, *p* < 0.01)	[19]
dairy	CH_4_/DMI (g/kg DMI) = −0.073 (±0.0084) × 3-NOP + 20.068 (±1.16) (R^2^ = 0.92, n = 16, *p* < 0.01)	[19]
long-term	CH_4_/DMI (g/kg DMI) = −0.053 (±0.0055) × 3-NOP + 21.379 (±2.11) (R^2^ = 0.91, n = 19, *p* < 0.01)	[19]
all	CH_4_/DOM (g/kg DOM) = −54.6 (±13.3) × 3-NOP + 30.6 (±1.32) (R^2^ = 0.68, n = 10, *p* < 0.01)	[16]
all	CH_4_/milk (g/kg milk) = −29.5 (±11.9) × 3-NOP + 14.0 (±1.90) (R^2^ = 0.46, n = 12, *p* < 0.05)	[16]
all	CH_4_/BW (g/kg BW) = −0.94 (±0.19) × 3-NOP + 0.486 (±0.04) (R^2^ = 0.42, n = 39, *p* < 0.01)	[16]
all	CH_4_ (g/d) = −0.00176 (±0.000411) × 3-NOP + 12.3 (*p* < 0.05)	[17]
all	CH_4_ (% of GEI) = −10.3 × 3-NOP + 6.16 (R^2^ = 0.49, n = 29, *p* < 0.01)	[16]

Note: ^1^ All refers to a combined dataset for beef, dairy and sheep and long-term refers to duration of feeding period in in vivo studies [14,49,50,51,55]; ^2^ 3-NOP = 3-nitrooxypropanol; BW = body weight; DMI = dry matter intake; DOM = digested organic matter; GEI = gross energy intake.

**Table 2:** Summary of 3-nitrooxypropanol (3-NOP) effects on in vivo fermentation, digestibility, microbes and enteric CH_4_ production in ruminants.

Reference	Animal	Diet and Level ^1^	3-Nitrooxypropanol (3-NOP)		Effects ^4^						
			mg/kg DM ^2^	Length of Experimental Period ^3^	VFA	Ammonia Nitrogen	CH_4_ Yield ^5^	CH_4_ Measurement	H_2_ Production	Digestibility ^6^	Microbes ^6^
Haisan et al. [32]	Dairy	Silage: concentrate (60:40)	130	28-d periods	↓ acetate and acetate-to-propionate ratio	NR	↓ (60% relative to a control diet)	Sulfur hexafluoride tracer technique	NR	NR	↓ Methanogens
Reynolds et al. [33]	Dairy	Silage: concentrate (51:49)	25 and 124	5-wk	↓ acetate and acetate-to-propionate ratio	–	↓ (7%, 9.8% relative to a control diet, g/d)	Respiration chambers	NR	↓ DM, OM, ADF, nitrogen, and energy by the higher dose	NR
Hristov et al. [14]	Dairy	TMR	40, 60, and 80	12-wk	NR	NR	↓ (25%, 31%, 32% relative to a control diet, g/d)	GreenFeed system	↑ 0.48, 0.96, and 1.27 g/d, respectively	NR	NR
Lopes et al. [46]	Dairy	Forage: concentrate (55:45)	60	Two 14-d periods	↓ acetate and acetate-to-propionate ratio	↓	↓ (34%, relative to a control diet)	GreenFeed system	↑ 1.3 g/d	NR	↓ Ruminococcus and Clostridium spp.
Haisan et al. [41]	Dairy	Silage: concentrate (60:40)	68 and 132	Three 28-d periods	↓ acetate	–	↓ (23–37% relative to a control diet)	Sulfur hexafluoride tracer technique	NR	↑ DM, NDF at high dose	# Methanogens, protozoa, and bacteria
Van Wesemael et al. [50]	Dairy	Silage: concentrate (66:34)	75 ^7^	10-wk	NR	NR	↓ (21–23% relative to a control diet)	GreenFeed units	NR	NR	NR
Melgar et al. [36]	Dairy	Forage: concentrate (58:42)	60	15-wk	↓ acetate, total VFA	–	↓ (21%, relative to a control diet)	GreenFeed system	↑ 48-fold relative to control diets	↑ crude protein	NR
Melgar et al. [58]	Dairy	Forage: concentrate (60:40)	40, 60, 80, 100, 150, and 200	31 d	NR	NR	↓ (16–36%, relative to a control diet)	GreenFeed system	↑ 6- to 10-fold relative to control diets	NR	NR
d 3 *ante partum* until 115 DIM	Dairy	Forage: concentrate (60:40)	51		NR	NR	↓ (17%, relative to a control diet)	Climate respiration chambers	↑ 11-fold	↑ DM, OM, NDF and gross energy	NR
Meale et al. [59]	Dairy	Milk and concentrate	3 mg/kg BW	14-wk	No effect	NR	↓ (11.6–17.5% relative to control calves, g/d)	GreenFeed system	NR	NR	↓ rumen bacteria and archaeal at 60 weeks of age
Melgar et al. [60]	Dairy	Forage: concentrate (58:42)	60	15-wk	NR	NR	↓ (27%, relative to a control diet)	GreenFeed units	↑ 6-fold relative to control diets	NR	NR
Pitta et al. [42]	Dairy	TMR	60	12-wk	NR	NR	NR	NR	NR	NR	↓ Methanobrevibacter, Methanosphaera
Schilde et al. [37]	Dairy	Silage: concentrate (90:10)	48 and 51	d 28 *ante partum* until d 120 *post-partum*	↓ acetate and acetate-to-propionate ratio	↓	↓ (23–35% relative to a control diet)	GreenFeed system	NR	NR	# protozoa
Romero-Perez et al. [40]	Beef	Forage: concentrate (60:40)	47, 144 and 305	Four 28-d periods	↓ acetate, acetate-to-propionate ratio	–	↓ (4–33%, relative to a control diet)	Whole animal metabolic chambers	NR	#	# Methanogens, protozoa, and bacteria
Romero-Perez et al. [49]	Beef	Forage: concentrate (60:40)	280	112 d	↓ acetate, acetate-to-propionate ratio	–	↓ (59.2%, relative to a control diet)	Whole animal metabolic chambers	NR	NR	↓ methanogens
Vyas et al. [51]	Beef	Silage: concentrate (70:30,8:92)	100 and 200	238 d	NR	NR	↓ (16–22.9% relative to a backgrounding control diet; 25.8–45.2% relative to a finishing control diet)	Open-circuit calorimetry Chambers	↑ 2.6- to 5.5-fold (backgrounding phase); 140- to 621.5-fold (finishing phase) relative to control diets	NR	NR
Vyas et al. [53]	Beef	Silage: concentrate (65:35,8:92)	50, 75, 100, 150, and 200	Two 28-d periods	NR	NR	↓ (max. 23% and 45% relative to high-forage and high-grain control diets)	Open-circuit calorimetry chambers	↑ max. 1.03 and 2.77 g/d.animal	NR	NR
Martínez-Fernández et al. [43]	Beef	grass hay	325	21 d	↓	↑	↓ (38%, relative to a control diet)	Open-circuit respiration chambers	–	↑DM	↓ Methanobrevibacter
Vyas et al. [55]	Beef	Silage: concentrate (65:35,8:92)	125 and 200	105 d	↓ acetate and acetate-to-propionate ratio	–	↓ (37–42% relative to a control diet)	Open-circuit calorimetry chambers	↑ 2.26 and 7.92 g/animal per day	NR	NR
Kim et al. [61]	Beef	Forage: concentrate (65:35)	100	Three 21-d periods	↓ acetate	–	↓ (18%, relative to high forage control diet)	GreenFeed system	NR	NR	NR
McGinn et al. [56]	Beef	Barley silage: barley grain (92:8)	125	120 d	NR	NR	↓ (70%, relative to a control diet)	Centration ratio and inverse dispersion methods	NR	NR	NR
Samsonstuen et al. [62]	Beef	Forage: concentrate (78:22, 47:53, 62:38, 50:50)	100 and 237	34-wk	NR	NR	↓ (15% and 31% for British breed, 19% and 35 % for Continental breed, kg CO_2_ eq kg^−1^ carcass)	HolosNorBeef modle	NR	NR	NR
Zhang et al. [45]	Beef	Forage: concentrate (90:10)	150	12 d	NR	NR	↓ (53%, relative to a control diet) ^8^	Gas chromatography	↑ 780%	# ruminal fiber degradation	↓ Methanobrevibacter for barley silage
Alemu et al. [38]	Beef	Silage: concentrate (70:30)	150, 175, and 200	108 d	NR	NR	↓ (20%, 25%, and 21% relative to a control diet)	GreenFeed system	↑ 3.5-, 4-, 4-fold relative to control diets	NR	NR
Alemu et al. [54]	Beef	Forage: concentrate (8:92)	100, 125 and 150	Three 28-d periods	↓ acetate: propionate ratio	–	↓ (52%, 76%, and 63% relative to a control diet)	GreenFeed system	↑ 4.9-fold	NR	NR
Gruninger et al. [44]	Beef	Forage: concentrate (90:10)	200	Four 28-d periods	↑ propionate percentages	NR	↓ (28.2%, relative to a control diet)	Open-circuit calorimetry chambers	↑ 37-fold relative to control diets	NR	↓ Methanobrevibacter, Methanomicrobium, Methanomethylophilus
Zhang et al. [57]	Beef	Forage: concentrate (90:10)	200	Four 28-d periods	↓ acetate, total VFA concentration	–	↓ (31.6%, relative to a control diet)	Open-circuit calorimetry chambers	↑ 45-fold relative to control diets	↑ crude protein and starch digestibility	NR
Martínez-Fernández et al. [52]	Sheep	Alfalfa hay and oats (60:40)	111	30 d	↓ acetate and acetate-to-propionate ratio	–	↓ (26%, relative to a control diet)	Respiration chambers	NR	# DM	# Methanogenic archaea

^1^ Dietary level on a dry matter (DM) basis. ^2^ 3-NOP concentration in the substrate. ^3^ DIM = days in milk; d = day; wk = week. ^4^ ↑ = increase; ↓ = decrease; NR = not reported; DM = dry matter; OM = organic matter; NDF = neutral detergent fiber; ADF = acid detergent fiber. ^5^ CH_4_ yield = per kg of DM, otherwise stated in g CH_4_/d. ^6^ ↑ = increase; ↓ = decrease; # = no statistically significant effect. ^7^ mixed with the basal diet or incorporated into a concentrate pellet. ^8^ Rumen dissolved CH_4_, mmol/L.

**Table 3:** Summary of 3-nitrooxypropanol (3-NOP) effects on in vitro fermentation, digestibility, microbes and enteric CH_4_ production in ruminants.

Reference	Animal (Rumen Fluid)	Diet Substrate and Level ^1^	3-Nitrooxypropanol (3-NOP)		Effects ^3^						
			mg/g DM ^2^	Persistency Time	VFA	Ammonia Concentration	CH_4_ Yield	CH_4_ Measurement	H_2_ Production	Digestibility	Microbes
Romero-Perez et al. [63]	cattle	Silage: concentrate (10 g; 60:40)	0.5, 1 and 2	7 d	↑ except for acetate	–	↓ (74.6%, 84.2% and 86%, relative to a control diet) ^4^	gas chromatograph	↑ 2.6, 3.05, and 3.18-fold respectively	– DM and OM	↓ Methanogens in the solid phase
Romero-Perez et al. [64]	cattle	Silage: concentrate (10 g; 60:40)	0.2	7 d	NR	NR	↓ (71.5%, relative to a control diet) ^5^	gas chromatograph	↑1.7-fold relative to control diets	NR	↓ Methanogens in the solid phase
Guyader et al. [65]	cattle	Silage: concentrate (10 g; 60:40)	0.5	19 d	↓ acetate and isovalerate	↑	↓ (75%, relative to a control diet)	gas-liquid chromatography	↑ (81%, relative to a control diet)	↑ DM and OM	NR
Romero-Perez et al. [66]	cattle	Silage: concentrate (10 g; 10:90)	0.2	6 d	↓ acetate	–	↓ (77.7%, relative to a control diet) ^5^	gas chromatograph	↑ 2.3-fold relative to control diets	– DM	↓ Methanogens
Alvarez-Hess et al. [67]	cattle	Corn grain (0.5 g; 50%) and alfalfa hay (0.5 g; 50%)	0.08	24 h	↓ acetate-to-propionate ratio	–	↓ (44%, relative to a control diet) ^6^	gas chromatography	NR	– DM	NR
Schilde et al. [68]	cattle	Forage: concentrate (12 g; 70:30, 40:60)	0.07, 0.16, and 1.2	48 h	↓ acetate, *iso*-butyrate	↓	↓ (17–97%, relative to a control diet) ^7^	gas chromatography	27- and 6.2-fold relative to low- and high-concentrate diets	↑DM	NR
Martínez-Fernández et al. [52]	sheep	alfalfa hay and oats (0.5 g; 60:40)	8 and 16	24 h	↓ acetate-to-propionate ratio	NR	↓ (86.1% and 95.4% relative to a control diet) ^8^	gas chromatograph	NR	NR	NR

^1^ Dietary level on a dry matter (DM) basis. ^2^ 3-NOP concentration in the substrate; d = day, semi long-term studies. ^3^ ↑ = increase; ↓ = decrease; – = no statistically significant effect; NR = not reported; DM = dry matter; OM = organic matter. ^4^ CH_4_ yield = mL/g DM degraded. ^5^ CH_4_ yield = mL/d. ^6^ CH_4_ yield = mL. ^7^ CH_4_ yield = mL/g DM degraded. ^8^ CH_4_ yield = μmol.

## Data Availability

Not applicable.
